# Trends in Axillary Lymph Node Dissection After Mastectomy Among Patients With Limited Nodal Burden

**DOI:** 10.1001/jamanetworkopen.2024.59692

**Published:** 2025-02-13

**Authors:** Ton Wang, Tyler Jones, Samantha M. Thomas, Astrid Botty Van den Bruele, Laura H. Rosenberger, Akiko Chiba, Kendra J. Modell Parrish, Lesly A. Dossett, Jennifer K. Plichta, Susan McDuff, Maggie L. DiNome, E. Shelley Hwang

**Affiliations:** 1 Department of Surgery, Duke University Medical Center, Durham, North Carolina; 2 Duke Cancer Institute, Duke University, Durham, North Carolina; 3 Biostatistics and Bioinformatics, Duke University, Durham, North Carolina; 4 Department of Surgery, Michigan Medicine, Ann Arbor; 5 Department of Population Health Sciences, Duke University Medical Center, Durham, North Carolina; 6 Department of Radiation Oncology, Duke University Medical Center, Durham, North Carolina

## Abstract

**Importance:**

Current practices in axillary treatment for patients with breast cancer who receive a mastectomy for node-positive disease are unknown. For patients who receive postmastectomy radiotherapy (PMRT), the addition of axillary lymph node dissection (ALND) may result in significant overtreatment.

**Objectives:**

To evaluate trends in axillary treatment for patients with limited nodal metastases who receive a mastectomy and identify factors that can be targeted to reduce axillary overtreatment.

**Design, Setting, and Participants:**

A retrospective cohort study was conducted of patients identified from the National Cancer Database who received a diagnosis of breast cancer from January 1, 2012, to December 31, 2021. Patients included were women aged 18 years or older with clinical (c) T1-T2N0 breast cancer who underwent mastectomy with axillary staging with sentinel lymph node biopsy (SLNB) and/or ALND and had 1 to 2 positive lymph nodes. Patients who received neoadjuvant therapies were excluded. Statistical analysis was performed from December 2023 to July 2024.

**Exposure:**

Axillary management based on ALND and PMRT receipt: (1) ALND alone, (2) PMRT alone, (3) both ALND and PMRT, and (4) neither ALND nor PMRT.

**Main Outcomes and Measures:**

Axillary management strategies were evaluated, and clinicopathologic characteristics based on treatment type were compared with multivariable analysis.

**Results:**

In total, 62 332 patients were included (median age, 58 years [IQR, 48-68 years]; 82.2% with Charlson-Deyo comorbidity score 0). The proportion of patients who received ALND alone decreased from 47.1% to 17.6% from 2012 to 2021, while the percentage of patients who received PMRT alone increased from 9.8% to 36.8%. Overall, 21.3% of patients received treatment with both ALND and PMRT, with little change over time (from 21.7% in 2012 to 17.7% in 2021). Most patients (88.4%) who received both PMRT and ALND underwent ALND at the same operation as SLNB. Younger age (odds ratio [OR] per year increase, 0.98 [95% CI, 0.98-0.98]; *P* < .001), high-grade tumors (grade 2: OR, 1.18 [95% CI, 1.09-1.29]; *P* < .001; grade 3: OR, 1.34 [95% CI, 1.22-1.48]; *P* < .001), presence of lymphovascular invasion (OR, 1.26 [1.19-1.33]; *P* < .001), and larger tumor size (cT2 tumors compared with cT1: OR, 1.10 [95% CI, 1.03-1.17]; *P* = .004; upstaging to pathologic T3 tumors: OR, 2.29 [95% CI, 1.15-4.99]; *P* = .03) were associated with increased likelihood of concurrent treatment with ALND and PMRT.

**Conclusions and Relevance:**

In this retrospective cohort study of patients with breast cancer who received a mastectomy, a substantial proportion of those with 1 to 2 positive lymph nodes were treated with both ALND and PMRT. Delaying the decision for ALND until after multidisciplinary input may reduce overtreatment.

## Key Points

**Question:**

What proportion of patients with breast cancer and node-positive disease undergo axillary lymph node dissection (ALND), and has this proportion changed over time?

**Findings:**

In this retrospective cohort study of 62 332 patients who received a diagnosis of breast cancer from 2012 to 2021, there was a decrease in the use of ALND (from 47.1% to 17.6%) and a corresponding increase in rates of postmastectomy radiotherapy (PMRT; from 9.8% to 36.8%) for management of 1 to 2 positive lymph nodes. However, approximately one-fifth of patients continued to receive both ALND and PMRT.

**Meaning:**

This study suggests that a substantial proportion of patients with breast cancer with 1 to 2 positive lymph nodes who received a mastectomy underwent treatment with both ALND and PMRT and may be at risk of axillary overtreatment and resultant morbidity.

## Teaser

This cohort study of more than 62 000 patients with breast cancer evaluates trends in axillary treatment for those with limited nodal metastases who received a mastectomy and identifies factors that can be targeted to reduce axillary overtreatment.

## Introduction

Over the past 2 decades, management of the axilla for patients with breast cancer with axillary nodal metastases has evolved significantly, with an overall trend toward surgical de-escalation.^1^ Axillary overtreatment can result in substantial morbidity and long-term harms, such as lymphedema, secondary malignant neoplasms, reduced quality of life, and financial toxicity, without reducing the risk of breast cancer recurrence or improving patient survival.^2,3,4^ Consequently, eliminating unnecessary locoregional treatment can significantly improve cancer survivorship.

The ACOSOG Z0011 (American College of Surgeons Oncology Group Z0011) trial demonstrated that sentinel lymph node biopsy (SLNB) alone resulted in equivalent overall and disease-specific survival when compared with axillary lymph node dissection (ALND) for patients with limited nodal disease burden undergoing breast-conserving surgery and adjuvant radiotherapy.^5^ As a result, ALND has been deimplemented in this setting, with national data showing that less than 20% of patients undergoing upfront breast-conserving therapy with 1 to 2 positive axillary lymph nodes (LNs) receive ALND in the post–ACOSOG Z0011 trial era.^6^ However, surgical de-escalation of the axilla for patients undergoing mastectomy with positive axillary LNs has been less straightforward. Although the ACOSOG Z0011 trial excluded patients who underwent mastectomy, the AMAROS (10981-22023 Comparison of Complete Axillary Lymph Node Dissection With Axillary Radiation Therapy in Treating Women With Invasive Breast Cancer) and SENOMAC (Sentinel Node Biopsy in Breast Cancer: Omission of Axillary Clearance After Macrometastases) trials included patients undergoing mastectomy and confirmed the safety of ALND omission for patients with up to 3 positive axillary LNs.^7,8^ In particular, the AMAROS trial found equivalent cancer outcomes with axillary radiotherapy compared with ALND. However, the risk of lymphedema was higher with ALND, while the risk of secondary malignant neoplasms was higher with axillary radiotherapy.^7,9^

These changes in surgical axillary management have occurred in tandem with shifting opinions on the role of postmastectomy radiotherapy (PMRT) to the chest wall and regional LNs for patients with pathologic N1 (pN1) disease. A combination of retrospective and prospective data suggest that a locoregional and disease-specific survival benefit is associated with PMRT for these patients.^10,11,12^ Consequently, many national guidelines recommend PMRT of the chest wall with intentional inclusion of the regional LNs for all patients with pN1 disease.^13,14^

For patients with limited nodal disease who receive PMRT, data support that the addition of ALND likely results in overtreatment of the axilla and significantly increases the risks of adverse effects, including lymphedema, without improving oncologic outcomes.^3,15^ The current National Cancer Center Network (NCCN) guidelines reflect this concern and recommend either ALND or PMRT, but not both, for patients with 1 to 2 positive axillary LNs after mastectomy and SLNB.^14^ In line with these recommendations, recent data from the Netherlands have shown a national shift away from ALND and toward PMRT and SLNB alone for the treatment of the axilla in patients with pN1 disease receiving a mastectomy.^16^ However, practice patterns in the US may differ significantly from those in the Netherlands given greater heterogeneity in the US of both patients and health care delivery systems, as well as historical difficulty in de-escalating care, even when new data suggest that a particular treatment strategy is not beneficial.^6,17^ Given this background, the objectives of this study are to (1) evaluate contemporary trends in axillary treatment for patients undergoing mastectomy with 1 to 2 positive axillary LNs and (2) evaluate patient, tumor, and facility-level factors that may be associated with these treatment choices to identify potential targets for deimplementation of axillary overtreatment in the US.

## Methods

### Data Source and Study Population

For this retrospective cohort study, data were selected from the National Cancer Database (NCDB), a registry of cancer cases from more than 1500 Commission on Cancer–accredited facilities in the US. Although the NCDB is not a population-based registry, it includes data on more than 80% of all breast cancer cases diagnosed nationwide.^18^ Given the deidentified nature of the data, the Duke University Health System institutional review board considered the study exempt and did not require informed consent. The study followed the Strengthening the Reporting of Observational Studies in Epidemiology (STROBE) reporting guideline.

We identified patients who received a diagnosis of invasive breast cancer from January 1, 2012, to December 31, 2021. Women aged 18 years or older with clinical (c) T1-T2N0M0 disease who underwent mastectomy with surgical axillary staging and were found to have 1 to 2 positive LNs were included. Individuals who did not receive their care at the reporting hospital, received neoadjuvant systemic therapy, or had missing pathologic nodal stage were excluded.

### Variable Definitions

Nodal positivity was defined as the presence of regional nodal micrometastases (0.2-2.0 mm) or macrometastases (>2.0 mm); patients with isolated tumor cells were not considered to have node-positive disease. Surgical axillary staging was defined as SLNB and/or ALND, either at the same operation or subsequent operations. Patients were defined as having received PMRT if they received radiotherapy after surgery or intraoperatively. Granular data on radiation fields are not available through the NCDB. Patients were categorized into 4 groups based on axillary management strategy: (1) ALND alone (with or without SLNB without PMRT), (2) PMRT alone (SLNB and PMRT without completion ALND), (3) both ALND and PMRT (ALND with or without SLNB and PMRT), and (4) neither ALND nor PMRT (SLNB only, without ALND or PMRT). Tumor receptor status was defined based on estrogen receptor, progesterone receptor, and ERBB2 receptor status. Hormone receptor (HR)–positive disease was defined as estrogen receptor–positive and/or progesterone receptor–positive tumors. Facility volume was defined as the mean number of patients with breast cancer a facility reported annually. Patient race and ethnicity are captured in the NCDB as self-reported information.^19^

### Statistical Analysis

Statistical analysis was performed from December 2023 to July 2024. Patients were grouped by year of diagnosis and summarized by axillary management strategy to evaluate treatment trends. Patient demographics, disease characteristics, and treatment details were summarized for patients in all treatment groups. Differences were tested using the χ^2^ test and analysis of variance as appropriate. A mixed-effects logistic regression model was fit by maximum likelihood to estimate the facility-level variation in treatment using facility variables as fixed effects with individual facilities as a random effect. This model showed very low intrafacility correlation; therefore, we opted to perform adjusted modeling without mixed effects as the main analysis. Multivariable logistic regression models including patient-, tumor-, and facility-level variables were fit to identify factors associated with different treatments. Age, year of diagnosis, and facility volume were treated as continuous variables; all other variables were treated categorically. No adjustments were made for multiple comparisons. *P* values were from 2-sided tests, and results were deemed statistically significant at *P* < .05. All statistical analyses were conducted using R, version 4.2.2 (R Project for Statistical Computing).

## Results

### Overall Characteristics

There were 62 332 patients (median age, 58 years [IQR, 48-68 years]; 6.4% Hispanic, 4.3% non-Hispanic Asian, 9.7% non-Hispanic Black, 77.8% non-Hispanic White, and 1.9% other non-Hispanic race or ethnicity; 82.2% with Charlson-Deyo comorbidity score 0) who met inclusion criteria (Table). The most common histologic type was ductal (73.2%) and most patients had grade 2 tumors (53.3%). The most common tumor receptor subtype was HR positive and ERBB2 negative (64.8%), followed by HR positive and ERBB2 positive (6.2%), HR negative and ERBB2 negative (4.9%), and HR negative and ERBB2 positive (1.9%). Most patients had pathologic (p) T1 (43.9%) or pT2 (48.6%) tumors. Most patients had pN1 disease with macrometastases (73.2%), while 26.8% had nodal micrometastases only (pN1mi). Patients were most commonly treated in comprehensive community cancer programs (41.8%) or academic or research programs (30.0%). A total of 21.3% of patients received both ALND and PMRT, 22.1% received PMRT only, 32.4% received ALND only, and 24.2% received neither PMRT nor ALND. Most patients with HR-positive disease received endocrine therapy (89.6%) and approximately half of all patients received chemotherapy (52.7%). Full characteristics of the study cohort are shown in the Table.

**Table.** Characteristics of Women 18 Years or Older in the National Cancer Database Diagnosed From 2012 to 2021 With Clinical T1-T2N0M0 Invasive Breast Cancer Who Underwent Mastectomy With Axillary Staging and 1 to 2 Positive Lymph Nodes

Variable	Participants, No. (%)					*P* value^a^
	All (N = 62 332)	ALND and PMRT (n = 13 271 [21.3%])	Neither (n = 15 102 [24.2%])	PMRT alone (n = 13 754 [22.1%])	ALND alone (n = 20 205 [32.4%])	
Age, median (IQR), y	58 (48-68)	54 (46-64)	61 (50-71)	56 (47-66)	60 (49-70)	<.001
Race and ethnicity						
Hispanic	3965 (6.4)	939 (7.1)	889 (5.9)	824 (6.0)	1313 (6.5)	<.001
Non-Hispanic Asian	2654 (4.3)	639 (4.8)	571 (3.8)	622 (4.5)	822 (4.1)	
Non-Hispanic Black	6052 (9.7)	1422 (10.7)	1341 (8.9)	1174 (8.5)	2115 (10.5)	
Non-Hispanic White	48 469 (77.8)	10 005 (75.4)	12 033 (79.7)	10 875 (79.1)	15 556 (77.0)	
Non-Hispanic other^b^	1192 (1.9)	266 (2.0)	268 (1.8)	259 (1.9)	399 (2.0)	
Charlson Comorbidity Score						
0	51 207 (82.2)	11 160 (84.1)	12 249 (81.1)	11 546 (83.9)	16 252 (80.4)	<.001
1	8071 (12.9)	1606 (12.1)	1981 (13.1)	1596 (11.6)	2888 (14.3)	
≥2	3054 (4.9)	505 (3.8)	872 (5.8)	612 (4.4)	1065 (5.3)	
Histologic type						
Ductal	45 597 (73.2)	9627 (72.5)	11 105 (73.5)	9509 (69.1)	15 356 (76.0)	<.001
Lobular	15 525 (24.9)	3398 (25.6)	3671 (24.3)	4052 (29.5)	4404 (21.8)	
Other	1210 (1.9)	246 (1.9)	326 (2.2)	193 (1.4)	445 (2.2)	
Tumor receptor status						
HR negative, ERBB2 positive	1154 (1.9)	234 (1.8)	279 (1.8)	190 (1.4)	451 (2.2)	<.001
HR positive, ERBB2 negative	40 365 (64.8)	8411 (63.4)	9991 (66.2)	10 025 (72.9)	11 938 (59.1)	
HR positive, ERBB2 positive	3894 (6.2)	893 (6.7)	924 (6.1)	816 (5.9)	1261 (6.2)	
HR negative, ERBB2 negative	3036 (4.9)	749 (5.6)	709 (4.7)	542 (3.9)	1036 (5.1)	
Unknown	13 883 (22.3)	2984 (22.5)	3199 (21.2)	2181 (15.9)	5519 (27.3)	
Clinical T stage						
T1	35 543 (57.0)	6779 (51.1)	9348 (61.9)	7341 (53.4)	12 075 (59.8)	<.001
T2	26 789 (43.0)	6492 (48.9)	5754 (38.1)	6413 (46.6)	8130 (40.2)	
Pathologic T stage						
T0/is	117 (0.2)	25 (0.2)	31 (0.2)	22 (0.2)	39 (0.2)	<.001
T1	27 384 (43.9)	4684 (35.3)	7661 (50.7)	5249 (38.2)	9790 (48.5)	
T2	30 298 (48.6)	6931 (52.2)	6782 (44.9)	6958 (50.6)	9627 (47.6)	
T3	4063 (6.5)	1507 (11.4)	519 (3.4)	1421 (10.3)	616 (3.0)	
T4	349 (0.6)	93 (0.7)	81 (0.5)	85 (0.6)	90 (0.4)	
Unknown	121 (0.2)	31 (0.2)	28 (0.2)	19 (0.1)	43 (0.2)	
Pathologic N stage						
N1	45 615 (73.2)	12 158 (91.6)	7321 (48.5)	10 307 (74.9)	15 829 (78.3)	<.001
N1mi	16 717 (26.8)	1113 (8.4)	7781 (51.5)	3447 (25.1)	4376 (21.7)	
Lymphovascular invasion						
Present	22 453 (36.0)	5695 (42.9)	4574 (30.3)	5354 (38.9)	6830 (33.8)	<.001
Absent	32 531 (52.2)	6057 (45.6)	10 528 (57.6)	6938 (50.4)	10 844 (53.7)	
Unknown	7348 (11.8)	1519 (11.4)	1836 (12.2)	1462 (10.6)	2531 (12.5)	
Grade						
1	9688 (15.5)	1644 (12.4)	2720 (18.0)	2084 (15.2)	3240 (16.0)	<.001
2	33 244 (53.3)	6936 (52.3)	8244 (54.6)	7569 (55.0)	10 495 (51.9)	
3	17 543 (28.1)	4319 (32.5)	3695 (24.5)	3765 (27.4)	5764 (28.5)	
Unknown	1857 (3.0)	372 (2.8)	443 (2.9)	336 (2.4)	706 (3.5)	
Facility location						
Northeast	11 155 (17.9)	2528 (19.0)	2497 (16.5)	2375 (17.3)	3755 (18.6)	<.001
Midwest	15 835 (25.4)	3717 (28.0)	3784 (25.1)	3443 (25.0)	4891 (24.2)	
South	23 869 (38.3)	4727 (35.6)	5971 (39.5)	5010 (36.4)	8161 (40.4)	
West	11 326 (18.2)	2273 (17.1)	2809 (18.6)	2874 (20.9)	3370 (16.7)	
Unknown	147 (0.2)	26 (0.2)	41 (0.3)	52 (0.4)	28 (0.1)	
Insurance						
Private insurance	35 208 (56.5)	8250 (62.2)	7684 (50.9)	8487 (61.7)	10 787 (53.4)	<.001
Medicaid	4403 (7.1)	1119 (8.4)	987 (6.5)	943 (6.9)	1354 (6.7)	
Medicare	20 306 (32.6)	3302 (24.9)	5899 (39.1)	3788 (27.5)	7317 (36.2)	
Not insured	974 (1.6)	250 (1.9)	213 (1.4)	187 (1.4)	324 (1.6)	
Other government insurance	822 (1.3)	227 (1.7)	159 (1.1)	228 (1.7)	208 (1.0)	
Unknown	619 (1.0)	123 (0.9)	160 (1.1)	121 (0.9)	215 (1.1)	
Facility type						
Integrated cancer network	13 451 (21.6)	2855 (21.5)	3392 (22.5)	3042 (22.1)	4162 (20.6)	<.001
Academic or research program	18 726 (30.0)	4227 (31.9)	4325 (28.6)	3852 (28.0)	6322 (31.3)	
Comprehensive community cancer program	26 084 (41.8)	5390 (40.6)	6352 (42.1)	5912 (43.0)	8430 (41.7)	
Community cancer program	4070 (6.5)	799 (6.0)	1033 (6.8)	948 (6.9)	1290 (6.4)	
Unknown	1 (0.002)	0	0	0	1 (0.005)	
Facility volume, median (IQR), No. of cases/y	297 (160-469)	303 (164-490)	293 (158-464)	294 (158-448)	300 (161-494)	<.001
Patients with no high school degree, %						
≥9.1	23 701 (38.0)	4953 (37.3)	5730 (37.9)	4862 (35.3)	8156 (40.4)	<.001
<9.1	29 405 (47.2)	6286 (47.4)	7203 (47.7)	6721 (48.9)	9195 (45.5)	
Unknown	9226 (14.8)	2032 (15.3)	2169 (14.4)	2171 (15.8)	2854 (14.1)	
Median income, $						
≥57 857	34 521 (55.4)	7285 (54.9)	8402 (55.6)	7745 (56.3)	11 089 (54.9)	<.001
<57 857	18 468 (29.6)	3920 (29.5)	4508 (29.9)	3811 (27.7)	6229 (30.8)	
Unknown	9343 (15.0)	2066 (15.6)	2192 (14.4)	2198 (16.0)	2887 (14.3)	
Radiotherapy	27 025 (43.4)	13 271 (100)	0	13 754 (100)	0	<.001
Endocrine therapy^c^	50 922 (89.6)	11 443 (95.2)	11 630 (84.1)	12 240 (95.1)	15 609 (86.1)	<.001
Chemotherapy	32 873 (52.7)	9475 (71.4)	5587 (37.0)	7925 (57.6)	9886 (48.9)	<.001

Abbreviations: ALND, axillary lymph node dissection; HR, hormone receptor; N1mi, N1 micrometastases; PMRT, postmastectomy radiotherapy.

^a^ Continuous variables were tested with 1-way analysis of variance; categorical variables were tested with the Pearson χ^2^ test.

^b^ Includes Fiji Islander, Guamanian, Hawaiian, Melanesian, Pacific Islander, Polynesian, Samoan, Tahitian, Tongan, and other race or ethnicity not otherwise specified.

^c^ Endocrine therapy for patients with HR-positive disease only (N = 56 860 for all participants, n = 12 019 for ALND and PMRT, n = 13 836 for neither, n = 12 872 for PMRT alone, and n = 18 133 for ALND alone).

### Trends in Surgical Axillary Management

**Figure 1.**
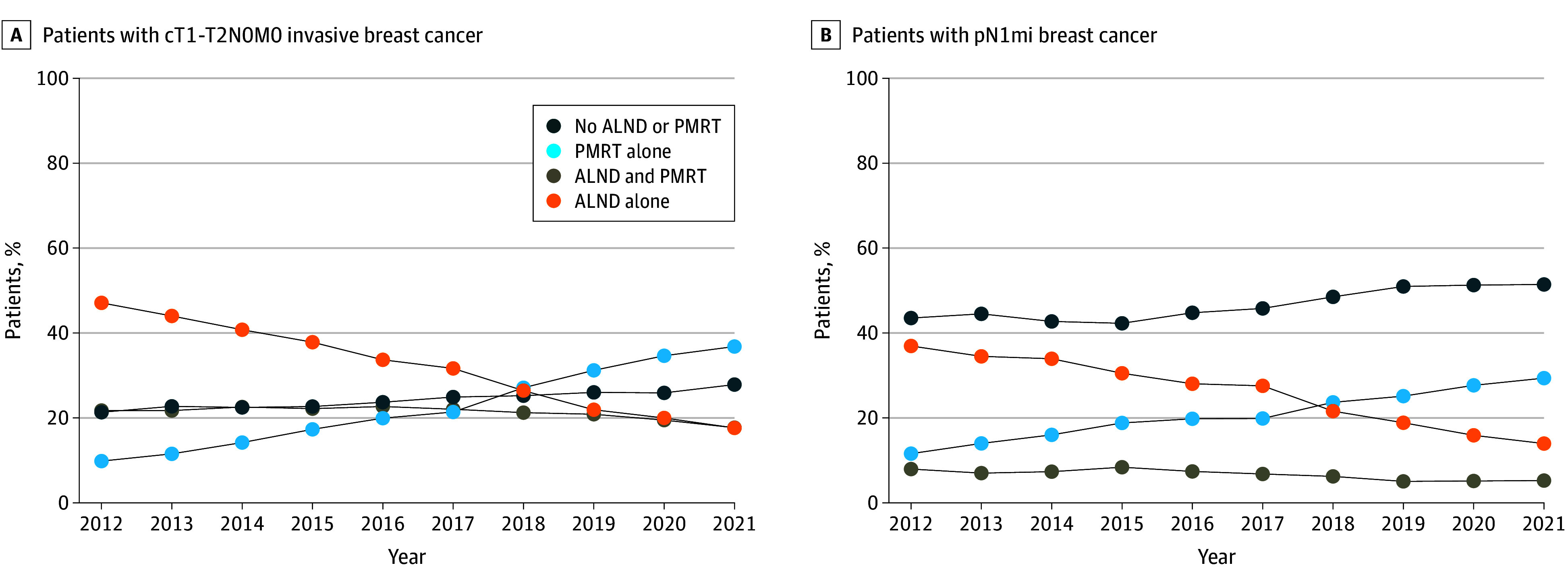
Trends in Axillary Management Strategies, 2012-2021 A, Trends in axillary management strategies for the entire cohort of women aged 18 years or older with a diagnosis of clinical T1-T2N0M0 (cT1-T2N0M0) invasive breast cancer who underwent mastectomy with axillary staging and had 1 to 2 positive lymph nodes. B, Trends in axillary management strategies for patients with micrometastatic disease (pN1mi) only. ALND indicates axillary lymph node dissection; PMRT, postmastectomy radiotherapy.

From 2012 to 2021, the percentage of patients receiving ALND alone steadily decreased from 47.1% to 17.6%, while the percentage of patients receiving PMRT alone steadily increased from 9.8% to 36.8%. There was a slight increase in patients receiving neither ALND nor PMRT, from 21.3% in 2012 to 27.9% in 2021, and a slight decrease in patients receiving both ALND and PMRT, from 21.7% in 2012 to 17.7% in 2021 (Figure 1). For patients with pN1mi, the percentage of patients receiving ALND alone decreased from 36.9% to 14.0%, while the percentage of patients receiving PMRT alone increased from 11.6% to 29.4% from 2012 to 2021. There was a slight increase in the percentage of patients receiving neither ALND nor PMRT, from 43.5% in 2012 to 51.4% in 2021, and 8.0% of patients in 2012 and 5.2% of patients in 2021 received both ALND and PMRT.

### Characteristics of Patients Receiving ALND Alone vs PMRT Alone

**Figure 2.**
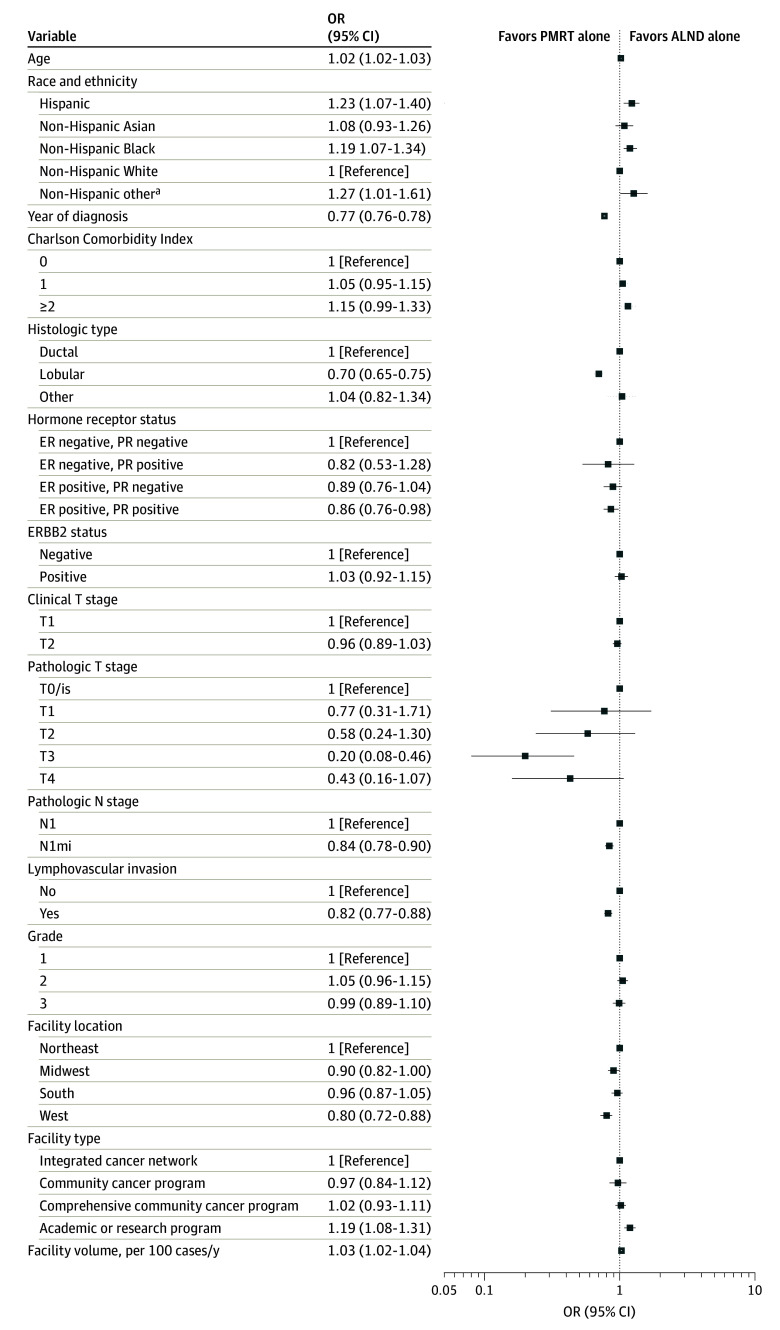
Multivariable Logistic Regression for Axillary Treatment With Axillary Lymph Node Dissection (ALND) Alone vs Postmastectomy Radiotherapy (PMRT) Alone Patients in the National Cancer Database were aged 18 years or older, received a diagnosis of clinical T1-T2N0M0 invasive breast cancer from 2012 to 2021, and underwent mastectomy with axillary staging and 1 to 2 positive lymph nodes. ER indicates estrogen receptor; N1mi, N1 micrometastases; OR, odds ratio; and PR, progesterone receptor. ^a^Includes Fiji Islander, Guamanian, Hawaiian, Melanesian, Pacific Islander, Polynesian, Samoan, Tahitian, Tongan, and other race or ethnicity not otherwise specified.

On multivariable logistic regression, patients who were older (odds ratio [OR] per year increase, 1.02 [95% CI, 1.02-1.03]; *P* < .001) and of non-Hispanic Black (OR, 1.19 [95% CI, 1.07-1.34]; *P* = .002) or Hispanic (OR, 1.23 [95% CI, 1.07-1.40]; *P* = .003) race and ethnicity compared with non-Hispanic White race and ethnicity, were at higher odds of receiving ALND alone vs PMRT alone (Figure 2). Patients with lobular compared with ductal histologic characteristics (OR, 0.70 [95% CI, 0.65-0.75]; *P* < .001) and with estrogen receptor–positive and progesterone receptor–positive compared with HR-negative tumors (OR, 0.86 [95% CI, 0.76-0.98]; *P* = .02) were at lower odds of receiving ALND rather than PMRT. Patients with upstaging to pT3 disease (OR, 0.20 [95% CI, 0.08-0.46]; *P* < .001), pN1mi compared with pN1 disease (OR, 0.84 [95% CI, 0.78-0.90]; *P* < .001), and tumors with lymphovascular invasion (LVI; OR, 0.82 [95% CI, 0.77-0.88]; *P* < .001) had significantly lower odds of receiving ALND alone compared with PMRT alone. Patients treated at facilities in the West compared with the Northeast (OR, 0.80 [95% CI, 0.72-0.88]; *P* < .001) and from regions with higher educational levels (OR, 0.88 [95% CI, 0.82-0.95]; *P* = .001) had lower odds of receiving ALND alone compared with PMRT alone, while patients treated at academic or research programs compared with integrated cancer network programs (OR, 1.19 [95% CI, 1.08-1.31]; *P* < .001) and higher-volume programs (OR, 1.03 [95% CI, 1.02-1.04]; *P* < .001) had significantly higher odds of treatment with ALND alone compared with PMRT alone. Compared with those with private insurance, patients with Medicaid (OR, 1.18 [95% CI, 1.04-1.35]; *P* = .01) or Medicare insurance (OR, 1.13 [95% CI, 1.03-1.25]; *P* = .009) had higher odds of receiving ALND alone compared with PMRT alone, while patients with other government insurance (OR, 0.64 [95% CI, 0.48-0.85]; *P* = .002) were at lower odds.

### Characteristics of Patients Receiving Both ALND and PMRT vs All Other Axillary Management Strategies

**Figure 3.**
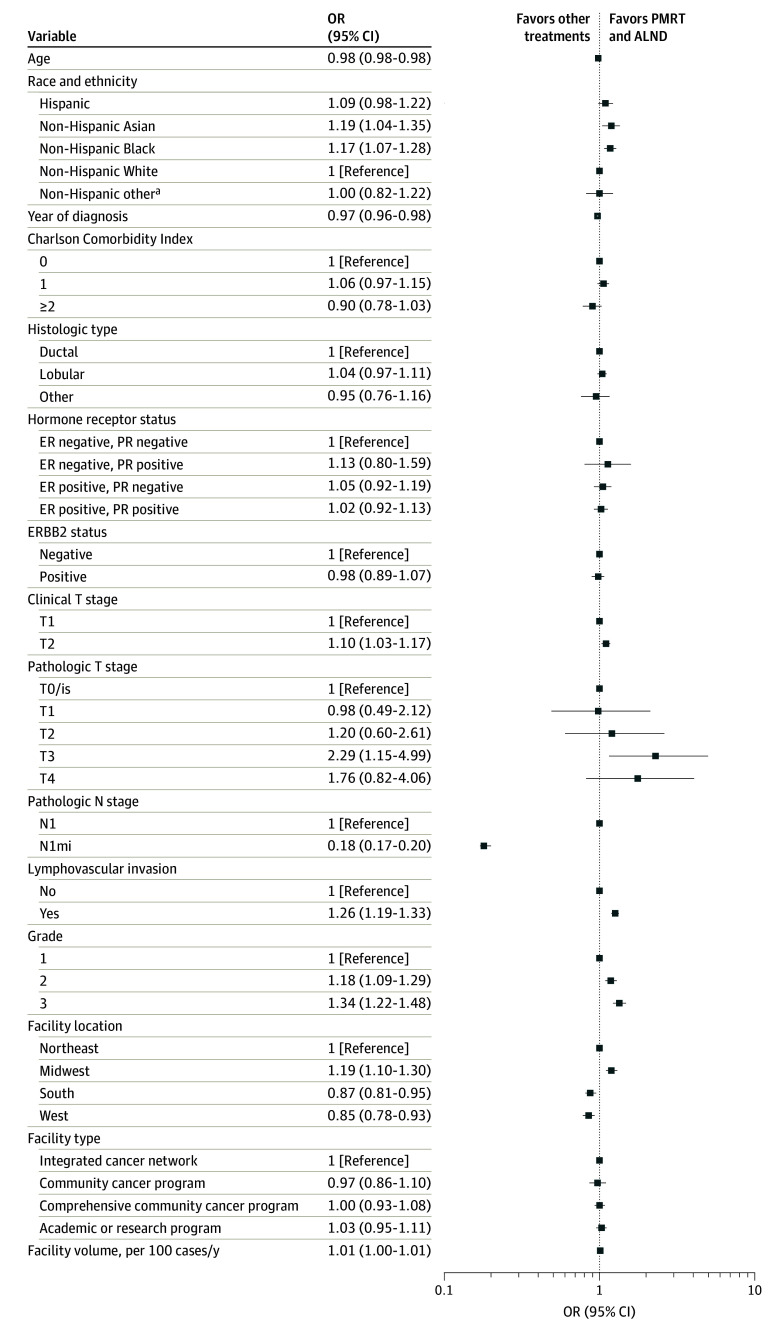
Multivariable Logistic Regression for Axillary Treatment With Both Axillary Lymph Node Dissection (ALND) and Postmastectomy Radiotherapy (PMRT) vs All Other Axillary Treatment Strategies Patients in the National Cancer Database were aged 18 years or older, received a diagnosis of clinical T1-T2N0M0 invasive breast cancer from 2012 to 2021, and underwent mastectomy with axillary staging and 1 to 2 positive lymph nodes. ER indicates estrogen receptor; N1mi, N1 micrometastases; OR, odds ratio; and PR, progesterone receptor. ^a^Includes Fiji Islander, Guamanian, Hawaiian, Melanesian, Pacific Islander, Polynesian, Samoan, Tahitian, Tongan, and other race or ethnicity not otherwise specified.

On multivariable logistic regression, patients who were younger (OR per year increase, 0.98 [95% CI, 0.98-0.98]; *P* < .001) and patients of non-Hispanic Asian (OR, 1.19 [95% CI, 1.04-1.35]; *P* = .009) or non-Hispanic Black (OR, 1.17 [95% CI, 1.07-1.28]; *P* = .001) race and ethnicity compared with non-Hispanic White race and ethniciy had significantly higher odds of treatment with both PMRT and ALND (Figure 3). Patients with cT2 tumors compared with cT1 (OR, 1.10 [95% CI, 1.03-1.17]; *P* = .004), upstaging to pT3 tumors (OR, 2.29 [95% CI, 1.15-4.99]; *P* = .03), higher-grade tumors (grade 2: OR, 1.18 [95% CI, 1.09-1.29]; *P* < .001; grade 3: OR, 1.34 [95% CI, 1.22-1.48]; *P* < .001), and tumors with LVI (OR, 1.26 [1.19-1.33]; *P* < .001) had significantly higher odds of treatment with both PMRT and ALND. Patients with pN1mi disease compared with pN1 (OR, 0.18 [95% CI, 0.17-0.20]; *P* < .001) had significantly lower odds of treatment with both PMRT and ALND. Patients with other government insurance compared with those with private insurance (OR, 1.30 [95% CI, 1.03-1.62]; *P* = .02), receiving treatment at facilities in the Midwest compared with the Northeast (OR, 1.19 [95% CI, 1.10-1.30]; *P* < .001), and receiving treatment at higher-volume facilities (OR, 1.01 [95% CI, 1.00-1.01]; *P* = .04) had significantly higher odds of treatment with both PMRT and ALND. Patients treated at facilities in the South (OR, 0.87 [95% CI, 0.81-0.95]; *P* = .001) or the West (OR, 0.85 [95% CI, 0.78-0.93]; *P* = .001) were at significantly lower odds of receiving both PMRT and ALND compared with those treated in the Northeast.

### Trends in Timing of ALND After SLNB for Patients Receiving Both ALND and PMRT

Of the patients who received SLNB followed by ALND and PMRT, 88.4% underwent ALND during the same operation as SLNB, while 11.6% underwent ALND in a subsequent operation. During the study period, there was a slight decrease in the proportion of patients undergoing SLNB and ALND during the same operation (89.1% of patients in 2012 vs 85.7% in 2021). These findings were similar for patients with pN1mi disease; 85.0% underwent ALND during the same operation as SLNB while 15.0% underwent ALND in a subsequent operation.

## Discussion

Our study shows a clear trend in the US toward de-escalation of ALND and a corresponding increase in the use of PMRT alone for the management of limited axillary nodal metastases for patients receiving a mastectomy. This substitution effect, in which clinicians are increasingly opting to treat axillary nodal disease with radiotherapy rather than surgical excision, appears to date back to at least 2011. A previous NCDB study of patients with breast cancer treated from 2006 to 2014 showed a trend toward decreased rates of ALND and increased rates of PMRT among patients with 1 to 2 positive lymph nodes undergoing mastectomy after publication of the ACOSOG Z0011 trial in 2011.^20^ Our findings in a more contemporary cohort of patients demonstrate continued acceptance of the safety of ALND omission in this patient population and are consistent with recently published data showing similar trends in the Netherlands.^16^ However, practice patterns in the US differ from those in the Netherlands in a notable way: our study demonstrates that approximately 20% of patients received axillary treatment with both ALND and PMRT, a proportion that remained consistent throughout the study period. In contrast, in the Netherlands, the proportion of patients treated with both ALND and PMRT declined significantly from 24% in 2009 to only 3% in 2018.^15^ Although we cannot directly compare the cohorts of patients in these studies, these results suggest that a much higher percentage of patients treated in the US compared with the Netherlands may be overtreated. Similarly, patients with pN1mi disease comprise a cohort in which aggressive axillary management is clearly not indicated. Although guidelines recommend against ALND for patients with pN1mi disease on SLNB, our results demonstrate that a substantial proportion of patients with pN1mi disease are receiving both ALND and PMRT.^14^ In the US, 14% of patients with pN1mi disease received ALND alone and 5% received both ALND and PMRT in 2021. In contrast, in the Netherlands only 0.4% of patients with pN1mi disease received ALND in 2018.^16^

Our study shows that patient and tumor characteristics, including younger age, higher tumor grade, presence of LVI, and larger tumor size, appear to be associated with an increased likelihood of concurrent treatment with both ALND and PMRT. Despite a bias that patients with these higher-risk tumor characteristics are at increased risk of locoregional recurrence and thus should be treated with both ALND and PMRT, there is little evidence to support a benefit associated with dual axillary treatment. The recent SENOMAC trial, which compared SLNB alone vs ALND for treatment of patients with 1 to 2 axillary macrometastases, did not show a benefit associated with ALND in reducing the risk of recurrence or death for any subgroup of patients, regardless of patient age, tumor size, surgery type, tumor subtype, or presence of extracapsular extension.^8^ However, the underrepresentation of certain high-risk subgroups in the SENOMAC trial, such as patients with T3 tumors, who comprised only 6% of trial participants, may be associated with clinician reluctance to de-escalate axillary treatment for these patients. The SENOMAC trial protocol deferred decision-making about PMRT and regional nodal irradiation to the treatment teams. As a result, approximately 90% of patients in the SENOMAC trial received adjuvant radiotherapy targeting the regional LNs, regardless of whether they underwent ALND.^8^ This finding suggests that multidisciplinary treatment teams are identifying an indication for nodal irradiation in nearly all patients with axillary nodal positivity, and the addition of ALND to nodal irradiation likely adds minimal benefit while posing the potential for significant harm.

In our study, 88.4% of patients who underwent SLNB followed by completion ALND and PMRT received SLNB and ALND during the same operation rather than undergoing separate operations. This finding suggests that many surgeons request intraoperative pathology consultation on the sentinel LNs and proceed with ALND if positive. Thus, one way to reduce axillary overtreatment is to avoid intraoperative frozen sections and defer any decisions about additional surgery until the final pathology report is available. This strategy has been proposed at Memorial Sloan Kettering Cancer Center; a recent institutional study found that intraoperative LN evaluation was associated with potentially avoidable ALND in 33% of patients who subsequently required PMRT.^21^ This finding is consistent with another study using the NCDB, which found that 41.0% of patients who received ALND after intraoperative pathologic evaluation received treatment with both ALND and PMRT.^22^ In comparison, only 4.9% of patients who did not receive intraoperative pathologic evaluation were subsequently treated with both ALND and PMRT. Overall, these studies suggest that most patients can avoid axillary overtreatment with ALND and PMRT if decision-making for ALND is deferred until the final pathology report is reviewed in a multidisciplinary setting. This strategy, in addition to minimizing the patient’s risk of lymphedema, can result in significant cost savings, as intraoperative frozen sections double the cost of pathologic examination.^23^

Our study showed associations between facility-level factors and axillary management strategy that were mostly not statistically significant; the associations that were statistically significant were not felt to be clinically meaningful or interpretable. Although there are some geographic variations that may be associated with heterogeneity in practice patterns, most factors associated with overtreatment are likely pervasive across facilities. As a result, strategies to reduce variation in axillary management will need to occur at the national level and could include increased dissemination of existing data and guidelines. For example, the current 2024 NCCN guidelines recommend consideration of no additional axillary surgery for patients after mastectomy with cT1-T2N0 disease with 1 to 2 positive sentinel LNs with planned adjuvant PMRT.^14^ However, this update did not occur until 2022 despite being based on data from the AMAROS trial, published in 2014,^9^ and the OTOASOR (Optimal Treatment of the Axilla—Surgery or Radiotherapy) trial, published in 2017.^24^ This lag between new trial data and subsequent incorporation into national guidelines may confuse clinicians as they attempt to discern how to maintain evidence-based practices.

Overall, the de-escalation of widely used cancer therapies in response to new evidence demonstrating that these therapies have limited benefit is extremely challenging, particularly in the US.^25,26,27^ As illustrated in our findings, there are frequently patient-, tumor-, clinician-, organization-, and country-specific factors associated with the decision to administer adjuvant cancer therapies. Our study provides meaningful information on how practice patterns in the US may differ substantially compared with other high-income countries. Although both the US and the Netherlands have adopted national initiatives, such as Choosing Wisely, to reduce overtreatment and low-value care, barriers to de-escalation differ significantly.^28^ A qualitative study of policymakers and experts on low-value care from the US, the Netherlands, and Canada found distinct cultural and health system differences between these high-income countries that may contribute to the findings of our study.^29^ The study notes that the US largely operates on a fee-for-service system compared with the Netherlands, where half of specialists are salaried, which may incentivize clinicians to provide more services. In addition, the study suggests that practitioners in the US are concerned about malpractice litigation and may be performing more defensive medicine compared with clinicians in the Netherlands, which has low malpractice payments compared with other countries. These factors are likely associated with the pace at which new evidence supporting de-escalation is incorporated into guidelines and clinician practice patterns.

### Strengths and Limitations

This study has some strengths; a significant strength is the large sample size, which allows for an updated examination of axillary treatment patterns in the US given that the NCDB represents approximately greater than 80% of all breast cancer cases diagnosed nationally. This study also has some limitations, including its retrospective nature and the imperfect availability of treatment data and tumor characteristics inherent to national cancer registries. Although data on PMRT are available in the NCDB, there is no information on specific fields for regional nodal irradiation. However, standard practice in the US for PMRT among patients with pN1 disease includes irradiation to the chest wall and the supraclavicular, infraclavicular, and axillary regional LNs, with or without inclusion of the internal mammary nodes. In addition, no data are available on the association of multidisciplinary influence or other patient- or clinician-level factors with axillary management decisions.

## Conclusions

Taken as a whole, our findings in this cohort study suggest that up to 20% of patients with early-stage breast cancer with limited axillary nodal metastases who undergo mastectomy are receiving both ALND and PMRT and may be at risk of overtreatment. We suggest that surgeons avoid intraoperative evaluation of sentinel LNs and engage their multidisciplinary colleagues, particularly in radiation oncology, before proceeding with ALND for positive sentinel LNs after mastectomy to minimize the long-term effect on quality of life that is associated with dual treatment with PMRT and ALND.

## Notes

Supplement.
Data Sharing Statement
