# Supplementary material for: Trends in Axillary Lymph Node Dissection After Mastectomy Among Patients With Limited Nodal Burden
Source: JAMA Netw Open. 2025 Feb 13;8(2):e2459692. doi: 10.1001/jamanetworkopen.2024.59692 (PMC11826356; doi:10.1001/jamanetworkopen.2024.59692)
Supplement: Supplement. — Data Sharing Statement [file jamanetwopen-e2459692-s001.pdf]

Data Sharing Statement

Wang. Trends in Axillary Lymph Node Dissection After Mastectomy Among Patients With Limited Nodal Burden. *JAMA Netw Open*. Published February 13, 2025. doi:10.1001/jamanetworkopen.2024.59692

Data
Data available: No

Additional Information
Explanation for why data not available: Data source is NDCB, all data available publicly
